# Effects of acute bouts of exercise and aerobic fitness on static balance in older adults

**DOI:** 10.1186/s13102-026-02226-2

**Published:** 2026-11-10

**Authors:** Arber Gashi, Tania Zieschang, Jessica Koschate-Storm

**Affiliations:** Faculty VI, Medicine and Health Sciences, Department of Health Services Research, Geriatric Medicine, Carl von Ossietzky Universität Oldenburg, Ammerländer Heerstr. 114-118, Oldenburg, 26129 Germany

**Keywords:** Fall risk, Aerobic fitness, Cardiorespiratory fitness, Balance, Physical exertion, Older adults

## Abstract

**Background:**

Falls are a major health concern in older adults and are closely linked to impaired balance. While regular physical activity improves health and helps prevent falls, acute bouts of exercise can transiently impair postural control. These exertion-related balance deficits have been linked to elevated lactate levels, which, in the context of physical exertion, may indicate insufficient oxygen delivery to working muscles. As aerobic fitness declines with age, older adults may be especially vulnerable to such impairments. This study aims to examine the effects of physical exertion on balance in older adults (1), assess whether aerobic fitness modulates post-exercise balance impairments (2), and explore group differences based on fall history (3).

**Methods:**

Overall, 105 community-dwelling older adults were enrolled (50, no fall history; 55, with fall history). Participants attended three laboratory visits, involving cardiopulmonary exercise testing to assess aerobic fitness and cardiorespiratory adaptability, and static balance assessments using inertial measurement units. Sway parameters were evaluated before and after treadmill exercise at or above the first ventilatory threshold (VT_1_). Mixed-model repeated-measures ANOVAs were used to test for group and condition effects.

**Results:**

Exercise at the level of VT_1_ significantly increased postural sway across multiple parameters, indicating a temporary deterioration of balance (*p* < 0.001 for sway area, sway range, RMS sway and sway path length). Aerobic fitness markers were not significantly associated with the magnitude of the immediate exercise-induced change in postural sway although heart rate adaptability was associated with better balance performance both before and after exercise exceeding the VT_1_ (*p* < 0.05 for eight out of ten sway parameters). No overall group differences were observed in postural sway or aerobic fitness markers. Participants with and without a fall history did not differ in overall postural sway or aerobic fitness, but showed different postural response patterns across the subsequent measurements.

**Conclusions:**

The observed increase in postural sway following exercise may indicate a transient period of reduced balance stability, although its implications for actual fall risk immediately after exertion remain uncertain. The findings suggest that cardiovascular adaptability may be associated with balance regulation. Future studies should include more heterogeneous study populations to better capture variability in aerobic fitness and functional outcomes.

**Trial registration:**

The local ethics committee approved the use of the experimental procedures (No. 2021–026), and the study was registered at the German Clinical Trials Register (DRKS00024890, date of registration: 28 January 2022).

**Supplementary Information:**

The online version contains supplementary material available at https://doi.org/10.1186/s13102-026-02226-2 .

## Background

Falls are a leading cause of injury and mortality among older adults, posing a significant public health concern. As the world population ages, projected to reach a proportion of 16% above the age of 65 years by 2050 [40], developing and refining preventive strategies becomes increasingly vital to mitigate the burden on the healthcare system.

Among the numerous risk factors for falls, emerging evidence consistently links lower aerobic fitness to an increased likelihood of falling in older adults [17, 21, 24, 34, 43]. Despite this growing body of evidence, neither the World Falls Guidelines [25], nor the latest Cochrane review on falls prevention through exercise in older people living in the community [33] have addressed the potential role of individual aerobic fitness on fall risk in older adults. This suggests a gap regarding our understanding of how physiological resilience, particularly aerobic fitness, may influence balance abilities and fall susceptibility in an aging population. Furthermore, recent evidence indicates that cardiocirculatory disorders, especially those characterized by transient hemodynamic instability such as orthostatic hypotension and arrhythmias, are also associated with falls [5], suggesting that cardiovascular regulation together with aerobic fitness may jointly shape fall susceptibility in older adults.

Aerobic fitness refers to the capacity of the cardiopulmonary and circulatory systems to deliver oxygen to working tissues and of skeletal muscle to utilize it during sustained activity, typically quantified by cardiopulmonary exercise testing (CPET), returning parameters such as maximal oxygen uptake (V’O_2max_) and oxygen uptake at the ventilatory threshold (V’O_2_@VT_1_) [2]. These indices reflect integrated central (cardiac output, vascular function) and peripheral (oxygen extraction) components of systemic oxygen transport, which are known to decline with advancing age [36].

Evidence from experimental studies indicates that physical exertion may transiently impair postural control in older adults. In treadmill-based protocols, conducted with community-dwelling older adults, postural instability immediately after walking exercise was reflected in increased center-of-pressure displacement and sway velocity across different stance and sensory conditions [7, 26]. In a cycling-based protocol involving recreationally active older men, moderate exertion led to increased postural sway during quiet bipedal stance, which correlated positively with blood lactate levels, suggesting that metabolic strain contributes to transient balance impairments [35]. Additionally, findings from young athletes undergoing high-intensity anaerobic exercise indicated that rising lactate concentrations may impair proprioception, as joint positioning errors increased alongside lactate accumulation [31]. These findings raise the question of how age-related changes in aerobic fitness may lower the threshold at which such impairments are triggered.

At exercise levels above the anaerobic threshold, lactate begins to accumulate in the blood because its production exceeds the body’s ability to buffer and clear it effectively, which, in the context of physical exertion, may reflect insufficient oxygen availability relative to metabolic demand in the working muscles. While healthy young adults typically begin to accumulate lactate only at high exertion levels (approximately ten times their resting oxygen uptake), unfit individuals, leading a sedentary lifestyle, may reach their anaerobic threshold during activities requiring as little as four times their resting oxygen uptake – corresponding to walking at a normal pace of about 4–5 km · h^−1^ [41]. Given the age-related decline of maximal oxygen uptake capacity (V’O_2max_) and oxygen uptake at the anaerobic threshold (V’O_2_@VT_1_) [6, 9, 30, 36], older adults may thus experience impairments in balance control at mild physical activities such as walking or climbing stairs. Consequently, even low-intensity daily tasks may impose sufficient metabolic strain to challenge postural stability in older adults.

In addition to the assessment of the V’O_2_@VT_1_, ventilatory efficiency represents another key marker of aerobic fitness. The ventilatory equivalent for carbon dioxide output at the first ventilatory threshold (V’E/V’CO_2_@VT_1_) reflects the amount of ventilation required to eliminate a given amount of carbon dioxide with elevated values indicating ventilatory inefficiency and impaired gas exchange. Previous research has shown that V’E/V’CO_2_@VT_1_ is an important prognostic marker in both cardiopulmonary and cardiovascular disease [39] and is sensitive to circulatory and pulmonary limitations that may not be fully captured by oxygen uptake alone. Including this parameter alongside V’O_2_@VT_1_ and circulatory adaptability therefore allows a more comprehensive assessment of aerobic fitness in relation to balance performance in older adults. While in the literature the terms “aerobic capacity”, “aerobic fitness”, and “cardiorespiratory fitness” are frequently used interchangeably [32], the present study adopts the term “aerobic fitness” as a broader marker encompassing both gas exchange analytics (V’O_2_@VT_1_, V'E/V’CO_2_) as well as circulatory adaptability to alternating exertion levels.

While lactate accumulation marks one important pathway linking exertion to impaired balance, the underlying circulatory dynamics also deserve attention. Borghi-Ricardo et al. [4] compared young and older healthy men and found that older adults exhibited markedly lower aerobic fitness (lower peak oxygen uptake, reduced peak heart rate, diminished peak power output) alongside slower cardiac output and stroke volume kinetics during both the onset and recovery of exercise. These findings reflect a clear age-related decline in maximal oxygen uptake capacity and delayed central hemodynamic adjustment. Furthermore, Houghton et al. [15] highlighted the role of vascular aging for oxygen delivery by showing that higher arterial stiffness, quantified using the augmentation index, a measure of wave reflection in the arteries, was strongly associated with reduced peak cardiac output in older adults. This decline in cardiac output limits the heart’s ability to deliver oxygen-rich blood to working muscles during exertion. One contributing factor is increased left ventricular afterload, which refers to the elevated resistance the heart must overcome to pump blood into stiffened arteries. As a result, the cardiovascular system becomes less efficient at meeting the body’s oxygen demands during physical activity. This impaired oxygen delivery capacity may explain why older adults, particularly those with underlying vascular changes, are more prone to early reliance on anaerobic mechanisms for ensuring adequate muscle contraction. Complementing these physiological observations, cardiocirculatory disease burden in older adults has also been linked to impaired postural stability and falls. In Bourke et al. [5] systematic review and meta-analysis of 184 studies, the most significant associations were reported for disorders that can cause transient cerebral hypoperfusion or hemodynamic instability, such as orthostatic hypotension and atrial fibrillation. These findings indicate that with advancing age, both slowed central hemodynamic kinetics and impaired vascular compliance can amplify exercise-induced mismatches in oxygen delivery leading to muscular and systemic lactate accumulation, thereby contributing to transient post-exertional balance impairments.

In summary, prior research suggests that both reduced oxygen uptake capacity and impaired oxygen delivery shaped by circulatory factors, such as cardiac output and vascular function, may compromise balance in older adults. Specifically, reduced oxygen uptake promotes earlier anaerobic engagement and greater lactate build-up, whereas delayed circulatory adjustments further exacerbate this metabolic strain by limiting timely oxygen delivery. Investigating whether differences in aerobic fitness and circulatory adaptability exist between older adults with and without a fall history is therefore essential, as such differences could help explain why some individuals are more vulnerable to falling than others.

Therefore, the present study aims to assess the effects of acute submaximal exercise on balance performance in older adults (1), to examine whether aerobic fitness is related to the magnitude of post-exercise balance impairments (2), and to determine whether postural responses and markers of aerobic fitness differ between older adults with and without a history of falls (3).

## Materials and methods

### Participants

A total of 105 community-dwelling older persons living in Lower Saxony in Germany were recruited for testing. Participants were recruited through flyers distributed at local senior support centers, the university library, and regional fitness centers, as well as through a newspaper article. In addition, a substantial proportion of participants were enrolled via peer referral. Participants were recruited from 10 December 2021 to 13 March 2023. No separate follow-up period was conducted, as outcomes were assessed during the laboratory appointments within the repeated-measures design. Participants were classified in two groups: *n* = 50 without a history of falling (nFH) and *n* = 55 with a history of falling (wFH). A fall was defined in accordance with the ProFane taxonomy as “an unexpected event in which the participants come to rest on the ground, floor or lower level” [20]. To be classified in the group with a history of falling, participants must have had a fall in the past 12 months or have had an injurious fall with the necessity of in-hospital treatment such as a fracture within the past 5 years.

The following exclusion criteria were applied: body weight of > 135 kg, due to the maximum permissible load of the treadmill used for the exercise protocols, the inability to walk 30 min without help, orthopedic limitations (acute arthritis or newly implanted prosthetics), neurologic diseases limiting or excluding the ability to walk on a treadmill, cardiovascular disease (arterial hypertension of > 180/110 mmHg, recent myocardial infarction or unstable coronary heart disease), poorly treated diabetes mellitus, poorly treated chronic obstructive pulmonary disease, severe visual impairment, and the inability to give consent to participation in a research project. Only two participants who expressed interest in participating could not be enrolled (*n* = 1 - poorly treated chronic obstructive pulmonary disease, *n* = 1 – walking aids/inability to walk on a treadmill without using them).

### Testing procedure/study design

The local ethics committee approved the use of the experimental procedures (No. 2021–026), and the study was registered at the German Clinical Trials Register (https://drks.de/search/en/trial/DRKS00024890/; DRKS-ID: DRKS00024890, registration date: 28.01.2022). Participants gave their written informed consent prior to the tests. The study was conducted in accordance with the Declaration of Helsinki, including its latest amendments until 2013. The present analyses are based on a subset of outcomes collected within the larger study.

**Fig. 1:**
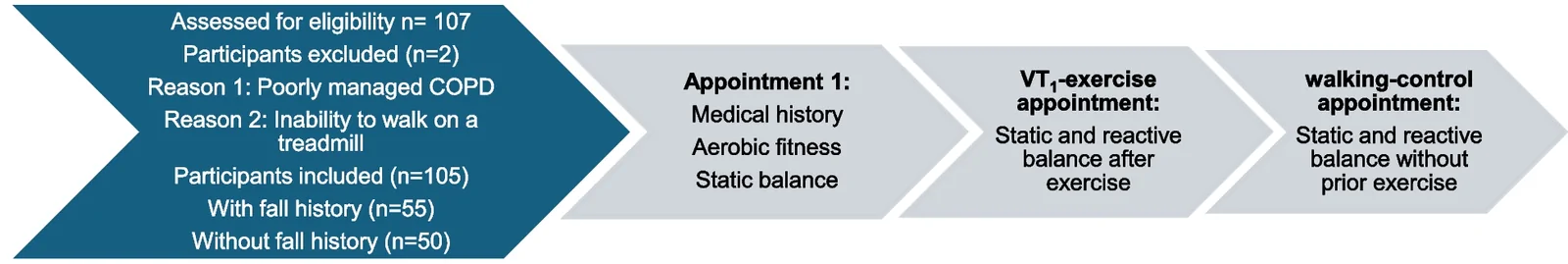
Flow chart of the study design. Participants completed one VT_1_-exercise appointment and one walking-control appointment in randomized order. The VT_1_-exercise appointment consisted of treadmill exercise at the individual first ventilatory threshold followed by two perturbed walking sessions, whereas the walking-control appointment consisted of the same two perturbed walking sessions without preceding VT_1_ exercise

This study was designed as an experimental mixed repeated-measures study with randomized order of exercise and walking-control appointments. The study is reported in accordance with the applicable items of the CONSORT guidelines. As the study did not involve randomized allocation of participants to intervention groups, but randomized the order of exercise and walking-control appointments, CONSORT items referring specifically to randomized group allocation were considered not applicable. Assessments were conducted over three appointments, each separated by at least one week (Fig. 1). The one-week interval was primarily chosen because participants wore an activPAL activity monitor for seven consecutive days between the first and second appointment. The activPAL data are not presented within this manuscript. All assessments were conducted in the Gait Lab at Carl von Ossietzky Universität​ Oldenburg, Oldenburg, Germany.

During the first appointment (Appointment 1), besides others, the overground walking speed, the preferred treadmill gait speed, aerobic fitness and static balance were assessed.

Notably, during Appointment 1, the exercise intensity to assess aerobic fitness was submaximal but exceeded the VT_1_ level.

During the second and third appointments, participants completed two structurally identical appointments that differed only with respect to the presence or absence of the individual VT_1_ exercise bout prior to the walking bouts with perturbations. One appointment was conducted as the exercise appointment, in which participants first performed the exercise protocol at the intensity corresponding to their individual VT_1_ (VT_1_-exercise appointment), followed by two standardized walking bouts with perturbations. The other appointment was conducted as the walking-control appointment, in which participants completed the same walking bouts with perturbations without the preceding VT_1_ exercise protocol. The order of the exercise and walking-control appointments was predetermined in randomized order, using block randomization with blocks of four and six participants.

### Aerobic fitness assessment

To adapt and individualize exercise intensity, participants first completed a standardized treadmill familiarization and speed calibration procedure [38] in the first appointment. Within the same session, aerobic fitness was assessed using a two-part exercise protocol on a treadmill (M-Gait Treadmill, Motek Medical B.V., Amsterdam, the Netherlands).

**Fig. 2:**
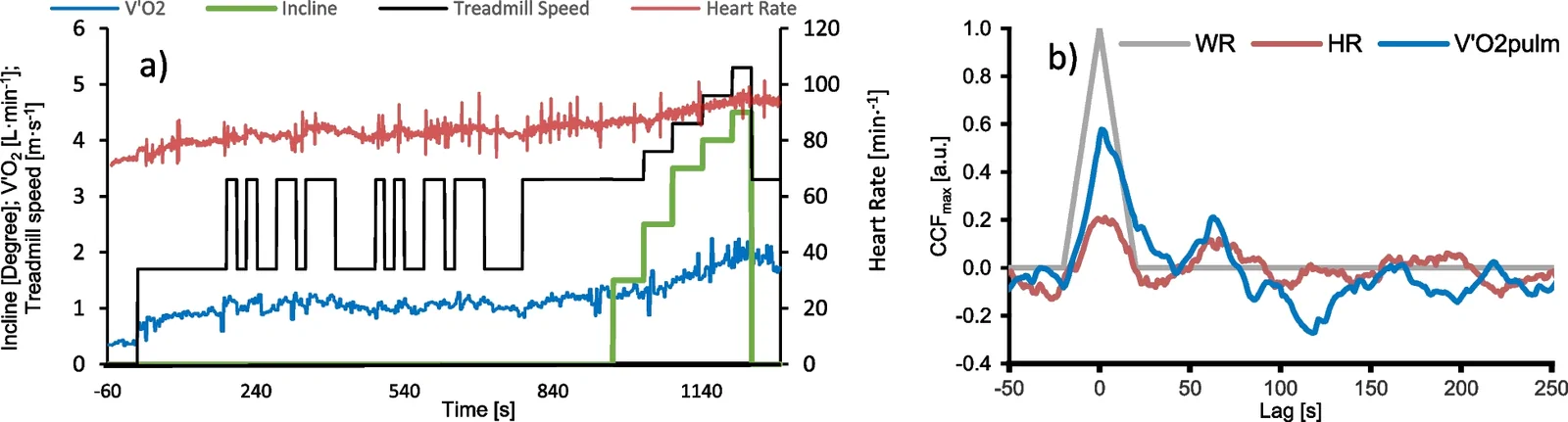
Graphic depiction of the treadmill exercise protocol in Appointment 1 (**a**) and the corresponding evaluation of the cardiorespiratory kinetics using cross-correlation functions (**b**). WR: work rate, HR: heart rate, V’O_2pulm_: pulmonary oxygen uptake, CCF_max_: maximum of the cross-correlation function between work rate and parameter x (HR or V’O_2pulm_)

First, participants completed a 10-min pseudo-random binary sequence (PRBS) protocol (Fig. 2), during which treadmill walking speed alternated between 50 and 100% of the individually preferred treadmill walking speed, without changes in treadmill incline [14, 19]. This phase was used to assess cardiorespiratory adaptability. For this purpose, cross-correlation functions (CCFs) were calculated between the treadmill speed signal and the physiological response signals, namely beat-to-beat heart rate and breath-by-breath pulmonary oxygen uptake. Thus, cardiorespiratory adaptability was derived from the dynamic coupling between the imposed PRBS treadmill speed changes and the corresponding heart rate and oxygen uptake responses over the PRBS phase. Before cross-correlation analysis, the physiological signals were synchronized with the treadmill protocol and interpolated to 1-s intervals to ensure homogeneous sampling. The treadmill speed signal was auto-correlated, resulting in the auto-correlation function ACF (speed), which was interpreted as the input impulse function of the imposed treadmill speed pattern. Heart rate and oxygen uptake were then cross-correlated with the treadmill speed signal, resulting in CCF_(HR)_ and CCF_(V’O2pulm)_, respectively. The maximum value of the CCF was extracted and reported separately as CCF_max_(HR) and CCF_max_(V’O_2pulm_). Higher CCF_max_ values indicate that the physiological response followed the imposed treadmill speed changes more closely and rapidly, reflecting better cardiorespiratory adaptability. Further methodological details of this time-series analysis approach are provided by [14].

Second, participants performed a ramp protocol with incremental increases in treadmill speed and incline every 60 s until the first ventilatory threshold (VT_1_) was exceeded. The ramp protocol was not intended to continue until the second ventilatory threshold (VT_2_) or maximal exhaustion. Rather, the aim was to identify and confirm the individual VT_1_, which was subsequently used to prescribe the individualized exercise intensity during the VT_1_-exercise appointment. To ensure a linear increase in work rate during the ramp protocol, in which both treadmill speed and incline were increased, we used the approach of Porszasz et al. [29]. Recovery after the exercise protocol was standardized with 60 s of walking at the preferred treadmill speed. An exemplary full exercise protocol and the analytic approach of the CCFs are illustrated in Fig. 2.

Aerobic fitness was assessed using cardiopulmonary exercise testing (CPET), combining breath-by-breath gas exchange analysis (CORTEX Biophysik GmbH, Leipzig, Germany), and heart rate (HR) monitoring via electrocardiogram (CustoBelt3, Custo med, Ottobrunn, Germany). The R-peaks of the ECG signal were extracted using a custom MATLAB (MATLAB, The MathWorks Inc., Natick, Massachusetts, United States, Version: R2022b) algorithm to derive beat-to-beat HR values, allowing for precise analysis of heart rate dynamics throughout the exercise protocol.

From the CPET data, V’O_2_@VT_1_ was determined using the V-Slope Method in combination with the respiratory exchange ratio and metabolic equivalents [3], providing a submaximal marker of oxygen adequacy for the given metabolic demand [37]. Ventilatory efficiency was further evaluated via the V’E/V’CO_2_@VT_1_, an established prognostic marker in cardiopulmonary and cardiovascular disease [39]. To capture cardiorespiratory adaptability, cross-correlation functions were calculated between the work rate signal and both HR (CCF_max_(HR)) and oxygen uptake (CCF_max_(V’O_2pulm_)) [14, 19], with higher values indicating faster adjustment of the respective parameter (kinetics).

The exercise protocol in the VT_1_-exercise appointment was designed to elicit physical exertion at each participant’s individually determined VT_1_. It consisted of three phases completed within eight minutes: 30 s of walking at 50% of the preferred treadmill speed, 90 s at the preferred speed, and six minutes at the treadmill speed and incline corresponding to each participant’s VT_1_, as determined during Appointment 1.

### Static balance assessment

Postural sway, as an indicator of static balance, was assessed on a firm surface, using inertial measurement units (Opal V1, Mobility Lab™ [ML], APDM, Inc., Portland, OR, USA). Measurements started after participants were instructed to stand still, feet close together, toes and heels touching and with hands at their hips while looking straight forward, for 30 s in different conditions.

**Fig. 3:**
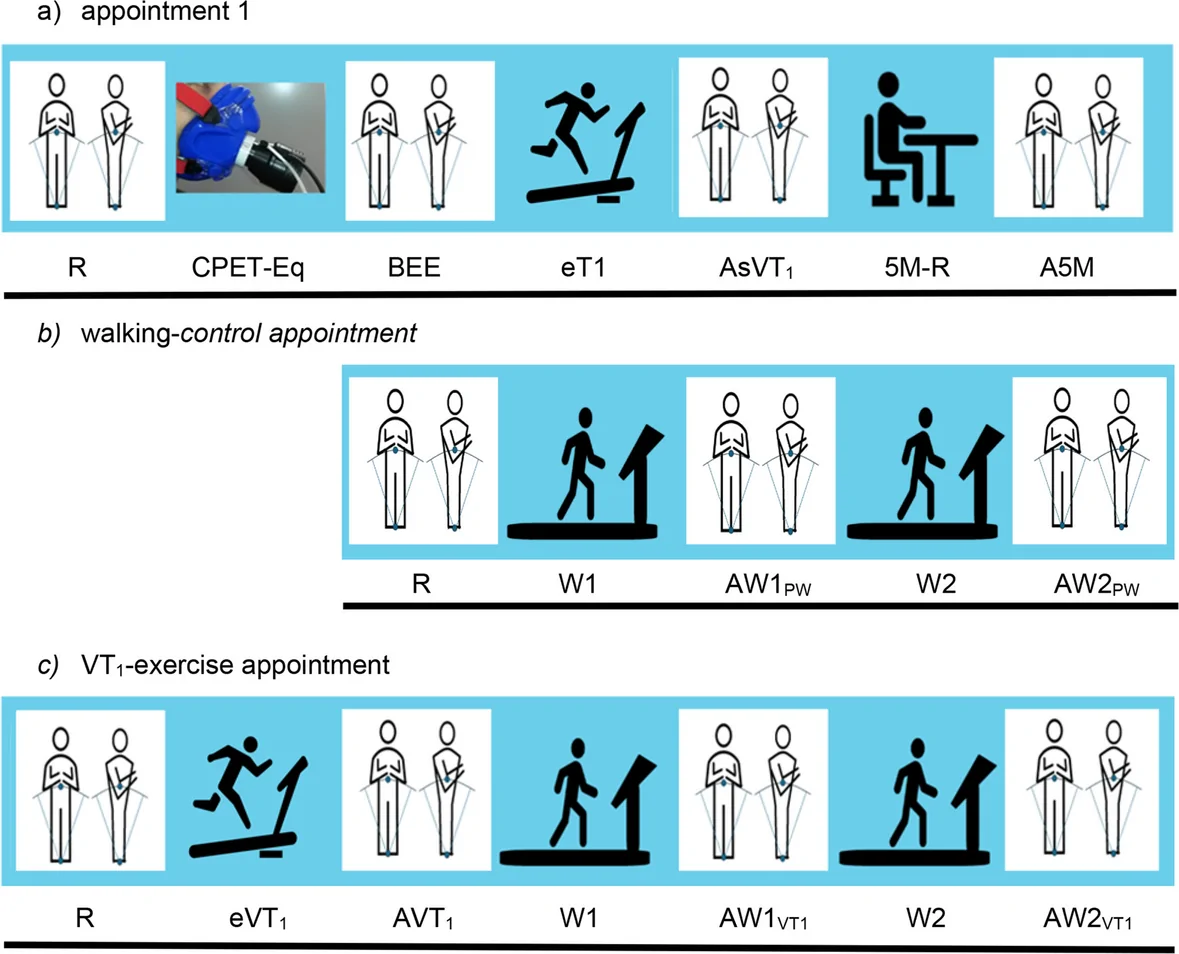
Graphic illustration of the testing procedure (**a**) appointment 1 (T1): R—resting, CPET-Eq – cardiopulmonary exercise testing equipment, BEE – sway before exercise with CPET equipment, eT1 – exercise protocol at appointment 1, AsVT_1_ – sway after exercise exceeding the first ventilatory threshold, 5 M-R – five minutes of rest—seated, A5M – sway after five minutes of rest; **b** walking-control appointment: R – resting, W1 – session one of perturbed walking, AW1_PW_ – sway after first session of perturbed walking, W2 – session two of perturbed walking, AW2_PW_ – sway after the second session of perturbed walking; **c** VT_1_-exercise appointment: R—resting, eVT_1_ – exercise protocol during the VT_1_-exercise appointment, AVT_1_ – sway after exercise at the level of the first ventilatory threshold, W1 – session one of perturbed walking, AW1_VT1_ – sway after first session of perturbed walking following VT_1_ exercise, W2 – session two of perturbed walking, AW2_VT1_ – sway after second session of perturbed walking following VT_1_ exercise

During the first appointment (T1), static balance was assessed four times: rest (R), before exercise while wearing cardiopulmonary testing equipment (BEE), immediately after the ramp protocol that exceeded the first ventilatory threshold (AsVT_1_), and after 5-min of seated rest (A5M). The measurements performed at T1 are illustrated in Fig. 3a.

During the second and third appointments, participants completed one walking-control appointment and one VT_1_-exercise appointment in randomized order. The two appointments were structurally identical except for the individualized exercise bout at the level of VT_1_, which was performed only in the VT_1_-exercise appointment (Fig. 3b and 3c).

In the walking-control appointment, static balance was measured at rest (R) and after two consecutive 4.5-min walking bouts with perturbations at the individually preferred treadmill gait speed (AW1_PW_ and AW2_PW_). Standardized gait perturbations were applied during both walking bouts according to the previously published SeFallED study protocol [38]. Static balance was measured immediately after each walking bout, with a two-minute standing rest period between walking trials.

In the VT_1_-exercise appointment, participants first performed six minutes of exercise at the intensity corresponding to their individual VT_1_. Static balance was measured at rest (R), immediately after six minutes of exercise at the level of the VT_1_ (AVT_1_), and after two consecutive 4.5-min walking bouts at the individually preferred gait speed (AW1_VT1_ and AW2_VT1_, respectively). The perturbation protocol, timing of the post-walking static balance assessments, and the two-minute standing rest period between walking trials were identical to those used in the walking-control appointment.

Reactive gait responses during perturbations are not the focus of the present investigation. However, because the same standardized perturbation protocol was applied in both the walking-control and the VT_1_-exercise appointments, the walking-control appointment served as the control condition. The measurements performed during the walking-control and VT_1_-exercise appointments are illustrated in Fig. 3b and c, respectively.

The parameters to evaluate postural sway for this study were selected based on their established reliability and sensitivity. Accelerometer-derived sway measures are strongly correlated with force-plate center of pressure metrics (*r* = 0.64–0.89 for key measures such as root mean square (RMS), path length, and sway area), which are established as gold standards for sway measures [22]. Test–retest reliability is reported as good (ICC = 0.81–0.89) for several time-domain parameters (sway path length 0.85–0.89, sway area 0.83–0.88, and mean velocity 0.81–0.86). More recently, McGough et al. [23] confirmed and extended this validation, reporting strong concurrent validity (*r* = 0.74–0.89) and high test–retest reliability (ICC = 0.79–0.92) across multiple sway parameters (path length, mean velocity, sway area, and RMS). These parameters were not interpreted as independent tests of balance, but as complementary quantitative indicators of static postural control. While sway area reflects the spatial extent of postural sway, sway range describes the maximal acceleration excursion, RMS sway reflects the overall magnitude of sway acceleration, mean velocity describes the average rate of sway movement, and path length reflects the cumulative sway trajectory. Together, these parameters provide a multidimensional description of postural sway during quiet standing.

Furthermore, O'Sullivan et al. [27] demonstrated the clinical relevance of these inertial metrics by showing that RMS sway acceleration was positively correlated with the TUG test (*r* = 0.54, *p* < 0.01) and inversely correlated with the SPPB score (*r* = − 0.49, *p* < 0.01), thereby linking quantitative sway assessment to established indicators of mobility and fall risk in older adults.

### Assessment of functional mobility

To assess general physical function, the Short Physical Performance Battery (SPPB), the Timed Up and Go (TUG) test, and the Short Falls Efficacy Scale-International (Short FES-I) were included in the testing protocol. The SPPB evaluates lower extremity function through balance, gait speed, and chair stand tasks, and is widely used as a predictor of disability, hospitalization, and mortality in older adults [11]. The TUG test measures the time required to stand up from a chair, walk three meters, turn and return to a seated position, serving as a simple and reliable screening tool for mobility, functional independence, and fall risk [28]. The Short FES-I, a self-report questionnaire, assesses concerns about falling across common daily activities, providing a validated measure of fall-related self-efficacy and psychological impact [18]. All three tests were performed at the end of the walking-control appointment.

### Statistical analysis

All statistical analyses were performed using IBM SPSS Statistics (Version 29.0.0.0 (241), IBM Corp., Armonk, NY). Analyses were conducted using available data for the respective outcome and measurement condition. Participants were analyzed according to fall-history classification and repeated-measures condition. Missing data were not imputed. Analyses were performed using available cases for each respective outcome and measurement condition. Normal distribution of the data was assessed using Shapiro–Wilk tests. Participant characteristics (e.g., age, BMI, preferred overground and treadmill walking speed) and functional mobility scores, including the SPPB, TUG, and Short FES-I were compared between the two groups of older adults wFH and nFH, using Mann–Whitney U-tests or independent t-tests, according to the respective data distribution. Differences in aerobic fitness parameters between the two groups, specifically oxygen uptake at the first ventilatory threshold (V′O_2_@VT_1_), ventilatory efficiency (V′E/V′CO_2_@VT_1_), and cardiorespiratory adaptability (CCF_max_(V’O_2pulm_) and CCF_max_(HR)) were compared similarly.

Balance outcomes derived from inertial sensors (sway area, acceleration range, RMS sway, mean velocity, and path length) were analyzed separately for each of the three testing appointments. Accordingly, three two-way mixed-model repeated measures ANOVAs were conducted, one for each appointment with conditions (Appointment 1: R, BEE, AsVT_1_, A5M; walking-control appointment: R, AW1_PW_, AW2_PW_; VT_1_-exercise appointment: R, AVT_1_, AW1_VT1_, AW2_VT1_) with group (wFH vs. nFH) as a between-subject factor. To directly compare the walking-control and VT_1_-exercise appointments, an additional mixed-model repeated-measures ANOVA was conducted using the matched time points only. In this model, appointment (walking-control vs. VT_1_-exercise) and condition (R, AW1, AW2) were included as within-subject factors, with group (wFH vs. nFH) as a between-subject factor. The matched post-walking measurements corresponded to AW1_PW_ and AW2_PW_ in the walking-control appointment and AW1_VT1_ and AW2_VT1_ in the VT_1_-exercise appointment. Main effects of Appointment, Condition, and Group, as well as the Appointment x Condition, Appointment x Group, Condition x Group, and the Appointment x Condition x Group interactions, were evaluated. For this analysis, R from the walking-control appointment and R from the VT_1_-exercise appointment were treated as the respective baseline measurements. AVT_1_ was not included in this analysis because no corresponding measurement at the same relative time point was available in the walking-control appointment. Sphericity was tested using Mauchly’s test, and Greenhouse–Geisser corrections were applied where appropriate. For all ANOVA models, post-hoc pairwise comparisons were adjusted using the Bonferroni correction.

To assess changes in balance after exercise, delta scores (Δ_sway_ = sway_AVT1_ – sway_R_) for the different balance outcomes (sway area, sway range, RMS sway, sway mean velocity, and sway path length) were calculated.

Finally, to explore potential associations between aerobic capacity and changes in postural control, Pearson and Spearman correlation analyses were performed between selected aerobic fitness parameters (e.g., V′O_2_/kg@VT_1_, V′E/V′CO_2_@VT_1_, CCF_max_(V’O2pulm), and CCF_max_(HR)) and post-exercise changes in sway measures (delta scores), according to data distribution. Pearson correlations were applied for normally distributed variables, whereas Spearman’s rank correlation coefficients were used for non-normally distributed variables. An alpha level of 0.05 was set to indicate statistical significance.

As the present analyses were based on a predefined subset of outcomes from a larger registered study, no separate a priori power calculation was performed for this specific analysis.

## Results

Participants’ characteristics are presented in Table 1. The two groups did not differ significantly in age, BMI, gait speed, treadmill speed, or SPPB score. However, participants of the wFH group reported higher concerns about falling and demonstrated lower functional mobility compared to the nFH group.

**Table 1:** Participant characteristics

	**Total, *n* = 105**	**wFH, *n* = 55**	**nFH, *n* = 50**	**group difference**
age [years] Median (25th/75th percentile)	72 (69.0/75.5)	71 (68/78)	72.5 (69/75)	*p* = 0.754
BMI [kg · m^−2^] average ± SD (min – max)	24.7 ± 3.2 (18–37)	24.4 ± 2.8 (18–31)	24.9 ± 3.5 (18—37)	*p* = 0.870
gait speed [km · h^−1^] average ± SD (min – max)	4.69 ± 0.8 (2.7–8.3)	4.57 ± 0.8 (2.7–8.3)	4.81 ± 0.9 (2.7–6.9)	*p* = 0.104
treadmill gait speed [km · h^−1^] average ± SD (min – max)	3.82 ± 0.7 (1.6–5.7)	3.7 ± 0.7 (2.5–5.4)	3.9 ± 0.7 (1.6–5.7)	*p* = 0.182
SPPB [score] Median (25th/75th percentile)	11.0 (10/12)	11.0 (9/12)	11.5 (10/12)	*p* = 0.396
Short FES-I [score] Median (25th/75th percentile)	8 (7/10)	9 (7/10)	8 (7/9)	***p* = 0.008**
Timed Up and Go [s] Median (25th/75th percentile)	8.1 (7.0/9.34)	8.3 (7.3/10)	7.5 (6.9/10.5)	***p* = 0.044**

Bold values indicate statistical​ly significant between-group differences (*p* < 0.05)

*SPPB* Short Physical Performance Battery, *Short FES-I* Short Falls Efficacy Scale International, *wFH* participants with a history of falling, *nFH* Participants without a history of falling

### Aerobic fitness

Results for cardiorespiratory fitness are summarized in Table 2. Of the 105 participants, two individuals from the wFH group were excluded from analyses of the Borg scale, speed@VT_1_, incline@VT_1_, V’O_2_@VT_1_, and V’E/V’CO_2_@VT_1_ due to a software malfunction during Appointment 1, which resulted in loss of data. Additionally, due to technical and synchronization issues, CCF_max_(HR) and CCF_max_(V’O_2pulm_) data from six participants from the wFH group could not be computed and were excluded from further analysis. Due to ECG-related artifacts, the data reporting of heart rate after 6 min of exercise at the first ventilatory threshold (HR@VT_1_) from 20 participants (*n* = 10 nFH, *n* = 10 wFH) were excluded from further analysis.

**Table 2:** Summary of the aerobic fitness parameters

	**All participants** **average ± SD** **(min–max)**	**wFH** **average ± SD** **(min–max)**	**nFH** **average ± SD** **(min–max)**	***P* value for group differences**
Borg Scale@VT_1_^#^ (6–20)	14 (13–15) *n* = 103	14 (13–16) *n* = 53	14 (13–15) *n* = 50	*p* = 0.567
Speed@VT_1_ [km⋅h^−1^]	4.6 ± 0.8 (2.1–6.4) *n* = 103	4.6 ± 0.7 (3.3–6.4) *n* = 53	4.7 ± 0.8 (2.1–6.4) *n* = 50	*p* = 0.349
Incline@VT_1_ [%]	6.6 ± 1.9 (2.0–13) *n* = 103	6.7 ± 1.8 (2.5–10) *n* = 53	6.4 ± 2.0 (2.0–13) *n* = 50	*p* = 0.402
Weber Classification	A, *n* = 101 B, *n* = 2 C, *n* = 0 D, *n* = 0	A, *n* = 53 B, *n* = 0 C, *n* = 0 D, *n* = 0	A, *n* = 48 B, *n* = 2 C, *n* = 0 D, *n* = 0	*p* = 0.143
HR@VT_1_ [beats · min^−1^]	125.52 ± 17.80 (89–164) *n* = 85	126.16 ± 18.43 (98–164) *n* = 45	124.69 ± 17.48 (98–165) *n* = 40	*p* = 0.709
V’O_2_@VT_1_ [ml⋅min^−1^⋅kg^−1^]	21.12 ± 3.69 (11.78–31.09) *n* = 103	20.95 ± 3.27 (14.78–28.36) *n* = 53	21.50 ± 4.16 (11.78–31.09) *n* = 50	*p* = 0.259
V’E/V’CO_2_@VT_1_ [a.u.]	30.1 ± 3.41 (22.8–39.2) *n* = 103	30.6 ± 3.68 (24.6–39.2) *n* = 53	29.6 ± 3.0 (22.8–37.9) *n* = 50	*p* = 0.144
CCF_max_(HR) [a.u.]	0.396 ± 0.193 (0.054–0.820) *n* = 99	0.377 ± 0.190 (0.079–0.725) *n* = 49	0.416 ± 0.195 (0.054–0.820) *n* = 50	*p* = 0.309
CCF_max_(V’O_2pulm_) [a.u.]	0.404 ± 0.114 (0.164–0.702) *n* = 99	0.406 ± 0.114 (0.164–0.618) *n* = 49	0.402 ± 0.115 (0.194–0.702) *n* = 50	*p* = 0.850

*Abbreviations V’O*_*2*_*@VT*_*1*_ Oxygen uptake at the first ventilatory threshold (103/105 valid measurements), *V’E/V’CO*_*2*_*@VT*_*1*_ Ventilatory equivalent for CO_2_ at the first ventilatory threshold (103/105 valid measurements), CCF_max_(HR) maximum of the cross-correlation function between work rate and heart rate (99/105 valid measurements), CCF_max_(V’O_2pulm_) maximum of the cross-correlation function between work rate and oxygen uptake (99/105 valid measurements), *Borg Scale@VT*_*1*_ Borg Scale after 6 min of exercise at the first ventilatory threshold (103/105 valid measurements), *HR@VT*_*1*_ Heart Rate after 6 min of exercise at the first ventilatory threshold (85/105 valid measurements), *Speed@VT*_*1*_ treadmill speed at the first ventilatory threshold (103/105 valid measurements), *Incline@VT*_*1*_ Treadmill incline at the first ventilatory threshold (103/105 valid measurements), *wFH* participants with a history of falling, *nFH* participants without a history of falling, a.u.: arbitrary units, Weber Classification (103/105 valid measurements) A: > 14 ml · min^−1^ · kg^−1^, B: 11–14 ml · min^−1^ · kg^−1^, C:8–11 ml · min^−1^ · kg^−1^, D: < 8 ml · min^−1^ · kg^−1^

^#^—median (25th – 75th percentile)

No significant group differences were observed in aerobic fitness parameters (V’O2@VT_1_, V’E/V’CO2@VT_1_, Borg Scale@VT_1_, CCF_max_(HR) and CCF_max_(V’O_2pulm_) and HR@VT_1_) between participants with and without a history of falls.

### Static balance

An overview of the static balance parameters (sway area, acceleration range, RMS, mean velocity, and path length of postural sway) across different conditions is presented in Tables 3, 4 and 5. Table 3 summarizes Appointment 1, Table 4 summarizes the VT_1_-exercise appointment, and Table 5 summarizes the walking-control appointment. Across the individual appointment analyses, condition effects were observed for several sway parameters, whereas significant main effects of Group and Condition x Group interactions were absent.

**Table 3:** Static balance parameters before and after exercise exceeding the first ventilatory threshold measured in appointment 1

	**R** **mean ± SD**			**BEE** **mean ± SD**			**AsVT**_**1**_ **mean ± SD**			**A5M** **mean ± SD**			**Condition**	**Group**	**Condition x Group**
	nFH	wFH	Total	nFH	wFH	Total	nFH	wFH	Total	nFH	wFH	Total			
**Sway area ** **[m**^**2**^**∙s**^**−4**^**]**	0.078 ± 0.055	0.070 ± 0.045	0.074 ± 0.050	0.159 ± 0.543	0.093 ± 0.058	0.124 ± 0.377	0.166 ± 0.164	0.143 ± 0.119	0.154 ± 0.142	0.086 ± 0.049	0.154 ± 0.296	0.122 ± 0.219	*p* = 0.102	*p* = 0.770	*p* = 0.195
**Acc. Range** **[m**^**2**^** · s**^**−2**^**]**	0.508 ± 0.206	0.485 ± 0.163	0.496 ± 0.184	0.631 ± 0.780	0.526 ± 0.156	0.576 ± 0.550	0.777 ± 0.487	0.671 ± 0.283	0.721 ± 0.395	0.521 ± 0.159	0.719 ± 0.856	0.625 ± 0.634	***p* = 0.007**	*p* = 0.867	*p* = 0.058
**RMS sway** **[m∙s**^**−2**^**]**	0.094 ± 0.030	0.089 ± 0.028	0.091 ± 0.029	0.113 ± 0.113	0.102 ± 0.032	0.107 ± 0.081	0.143 ± 0.073	0.130 ± 0.062	0.136 ± 0.068	0.099 ± 0.030	0.125 ± 0.101	0.112 ± 0.007	**p < 0.001**	*p* = 0.915	*p* = 0.075
**Mean velocity ** **[m∙s**^**−1**^**]**	0.184 ± 0.078	0.173 ± 0.079	0.179 ± 0.078	0.234 ± 0.238	0.191 ± 0.095	0.211 ± 0.179	0.250 ± 0.181	0.219 ± 0.109	0.234 ± 0.148	0.196 ± 0.079	0.227 ± 0.186	0.213 ± 0.145	***p* = 0.029**	*p* = 0.419	*p* = 0.199
**Path length ** **[m∙s**^**−2**^**]**	7.766 ± 2.787	8.169 ± 3.845	7.977 ± 3.373	8.347 ± 2.926	8.367 ± 2.975	8.357 ± 2.938	12.492 ± 5.029	12.704 ± 6.565	12.603 ± 5.857	8.680 ± 2.662	10.377 ± 8.645	9.569 ± 6.548	**p < 0.001**	*p* = 0.432	*p* = 0.325

Bold values indicate statistically significant effects (*p* < 0.05)

*Abbreviations: Acc* acceleration, *R* rest, *BEE* before exercise equipped, *AsVT*_*1*_ after exercise exceeding (supra – VT_1_) the first ventilatory threshold—equipped, *A5M* after 5 min of rest – not equipped, *RMS* root mean square, *wFH* participants with a history of falling, *nFH* – participants without a history of falling, *SD* standard deviation

**Table 4:** Static balance parameters before and after exercise at the level of the first ventilatory threshold measured during the VT_1_-exercise appointment

	**R** **mean ± SD**			**AVT**_**1**_ **mean ± SD**			**AW1**_**VT1**_ **mean ± SD**			**AW2**_**VT1**_ **mean ± SD**			**Condition**	**Group**	**Condition x Group**
	nFH	wFH	Total	nFH	wFH	Total	nFH	wFH	Total	nFH	wFH	Total			
**Sway area** **[m**^**2**^**∙s**^**−4**^**]**	0.073 ± 0.052​	0.068 ± 0.048	0.071 ± 0.050	0.104 ± 0.081	0.108 ± 0.071	0.106 ± 0.075	0.085 ± 0.087	0.097 ± 0.072	0.092 ± 0.079	0.088 ± 0.077	0.087 ± 0.052	0.087 ± 0.065	**p < 0.001**	*p* = 0.851	*p* = 0.501
**Acc. Range** **[m**^**2**^** · s**^**−2**^**]**	0.484 ± 0.146	0.466 ± 0.131	0.475 ± 0.138	0.647 ± 0.548	0.648 ± 0.354	0.647 ± 0.455	0.523 ± 0.232	0.564 ± 0.210	0.544 ± 0.221	0.526 ± 0.198	0.530 ± 0.163	0.528 ± 0.180	***p* = 0.001**	*p* = 0.849	*p* = 0.675
**RMS sway** **[m∙s**^**−2**^**]**	0.091 ± 0.030	0.088 ± 0.026	0.089 ± 0.028	0.109 ± 0.041	0.113 ± 0.036	0.111 ± 0.039	0.095 ± 0.038	0.108 ± 0.043	0.102 ± 0.041	0.100 ± 0.040	0.100 ± 0.030	0.100 ± 0.035	**p < 0.001**	*p* = 0.539	*p* = 0.135
**Mean velocity** **[m∙s**^**−1**^**]**	0.186 ± 0.095	0.173 ± 0.090	0.179 ± 0.092	0.192 ± 0.109	0.199 ± 0.086	0.196 ± 0.097	0.181 ± 0.096	0.218 ± 0.121	0.200 ± 0.111	0.212 ± 0.144	0.207 ± 0.111	0.209 ± 0.128	*p* = 0.071	*p* = 0.688	*p* = 0.064
**Path length** **[m∙s**^**−2**^**]**	8.031 ± 2.684	8.298 ± 3.668	8.169 ± 3.220	11.089 ± 3.698	11.885 ± 5.258	11.502 ± 4.571	8.928 ± 3.207	10.134 ± 4.930	9.554 ± 4.215	8.708 ± 2.907	9.507 ± 4.181	9.123 ± 3.630	**p < 0.001**	*p* = 0.277	*p* = 0.354

Bold values indicate statistically significant effects (*p* < 0.05)

*Abbreviations: Acc* acceleration, *R* Resting, *AVT*_*1*_ After exercise at the level of the first ventilatory threshold, *AW1*_*VT1*_ after the first session of perturbed walking following exercise at the level of the first ventilatory threshold, *AW2*_*VT1*_ after the second session of perturbed walking following exercise at the level of the first ventilatory threshold, *RMS* root mean square, *nFH* without fall history, *wFH* with fall history, *SD* standard deviation

**Table 5:** Static balance parameters before and after walking sessions in the walking-control appointment

	**R ** **mean ± SD**			**AW1**_**PW**_ **mean ± SD**			**AW2**_**PW**_ **mean ± SD**			**Condition**	**Group**	**Condition x Group**
	nFH	wFH	Total	nFH	wFH	Total	nFH	wFH	Total			
**Sway area** **[m**^**2**^**∙s**^**−4**^**]**	0.068 ± 0.042	0.067 ± 0.039	0.068 ± 0.040	0.068 ± 0.044	0.089 ± 0.062	0.079 ± 0.055	0.079 ± 0.059	0.095 ± 0.106	0.087 ± 0.087	***p* = 0.039**	*p* = 0.215	*p* = 0.270
**Acc. Range** **[m**^**2**^** · s**^**−2**^**]**	0.460 ± 0.131	0.468 ± 0.135	0.464 ± 0.133	0.479 ± 0.155	0.560 ± 0.283	0.522 ± 0.234	0.492 ± 0.149	0.533 ± 0.254	0.514 ± 0.211	***p* = 0.005**	*p* = 0.152	*p* = 0.147
**RMS sway** **[m∙s**^**−2**^**]**	0.087 ± 0.027	0.087 ± 0.024	0.087 ± 0.025	0.088 ± 0.028	0.100 ± 0.031	0.095 ± 0.030	0.094 ± 0.032	0.103 ± 0.047	0.099 ± 0.040	***p* = 0.005**	*p* = 0.170	*p* = 0.147
**Mean velocity** **[m∙s**^**−1**^**]**	0.184 ± 0.087	0.168 ± 0.086	0.176 ± 0.086	0.166 ± 0.080	0.188 ± 0.084	0.177 ± 0.082	0.183 ± 0.091	0.211 ± 0.125	0.198 ± 0.111	*p* = 0.117	*p* = 0.389	*p* = 0.132
**Path length** **[m∙s**^**−2**^**]**	7.606 ± 2.257	8.256 ± 3.270	7.950 ± 2.844	8.006 ± 2.791	9.608 ± 4.366	8.854 ± 3.778	8.274 ± 2.540	9.158 ± 4.514	8.742 ± 3.727	***p* < 0.001**	*p* = 0.097	*p* = 0.126

Bold values indicate statistically significant effects (*p* < 0.05)

*Abbreviations: Acc* Acceleration, *R* Resting, *AW1*_*PW*_ After the first session of perturbed walking, *AW2*_*PW*_ After the second session of perturbed walking, *RMS* Root mean square, *nFH* without fall history, *wFH* with fall history, *SD* Standard deviation

**Fig. 4:**
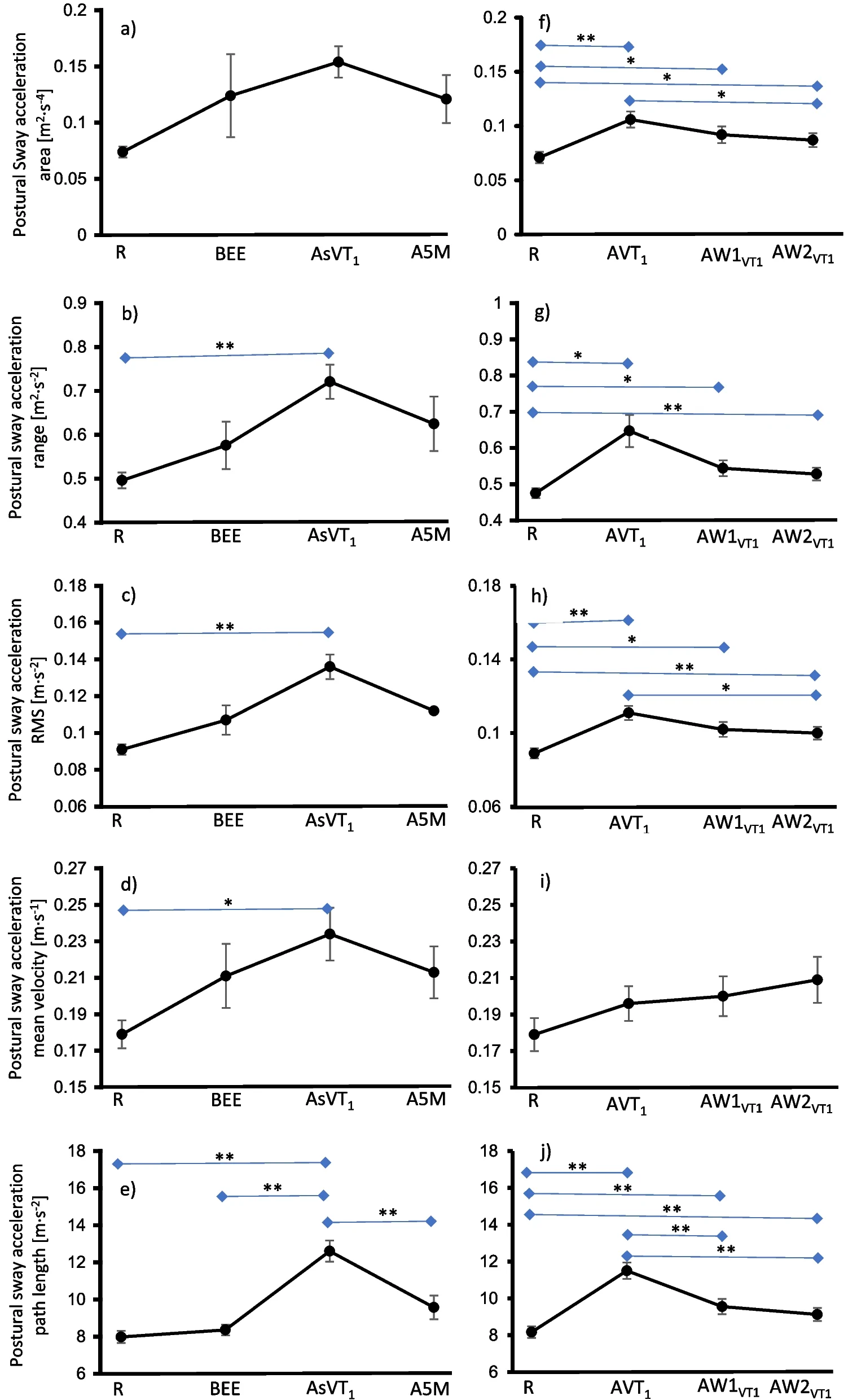
Different postural sway parameters under different conditions in Appointment 1 (**a**, **b**, **c**, **d**, and **e**) and VT_1_-exercise appointment (**f**, **g**, **h**, **i**, and **j**). *—*p* < 0.05 and **—*p* < 0.001

Figure 4 displays the changes in sway parameters over time for Appointment 1 and the VT_1_-exercise appointment, highlighting the time points at which significant post-hoc differences between conditions were observed. The corresponding graphical visualization of the walking-control appointment is provided in Supplementary Figure S2. Group-specific patterns are shown in Supplementary Figure S1 for Appointment 1 and VT_1_-exercise appointments and in Supplementary Figure S2 for the walking-control appointment.

The direct comparison between the walking-control and VT_1_-exercise appointments across matched conditions is reported in Supplementary Table S3. No significant Appointment x Condition interaction was observed for any sway outcome. Significant Condition x Group interactions were observed for sway acceleration range (*p* = 0.022), RMS sway (*p* = 0.006), mean velocity (*p* = 0.026), and path length (*p* = 0.012), whereas the interaction for sway area did not remain significant after correction for sphericity (*p* = 0.054). No significant Appointment x Condition x Group interaction was observed for any sway outcome.

The ∆_sway_ values for the immediate exercise-induced change during the VT_1_-exercise appointment are presented in Supplementary Table S1. No significant differences between participants without fall history (nFH) and with fall history (wFH) were observed.

### Relationship between aerobic fitness and static balance

The relationships between cardiorespiratory fitness and static balance parameters before and after exercise exceeding VT_1_ and exercise at the level of VT_1_ are listed in Table 6. Significant negative correlations were observed between heart rate adaptability (CCF_max_(HR)) and different sway measures before and after exercise. The correlations between aerobic fitness markers and Δ_sway_ for different balance parameters are presented in Table S2 provided in the Supplementary Material. Noteworthy, no significant correlations between aerobic fitness markers and Δ_sway_ were observed.

**Table 6:** Correlations between markers of aerobic fitness and postural sway parameters before and after exercise

		acc. sway area before [m^2^∙s^−4^]	acc. sway area after [m^2^∙s^−4^]	acc.range before [m^2^ · s^−2^]	acc.range after [m^2^ · s^−2^]	acc. RMS sway before [m∙s^−2^]	acc. RMS sway after [m∙s^−2^]	acc. mean velocity before [m∙s^−1^]	acc. mean velocity after [m∙s^−1^]	acc. path length before [m∙s^−2^]	acc. path length after [m∙s^−2^]
Exercise exceeding the VT_1_	**V’O**_**2**_**@VT**_**1**_ **[ml · min**^**−1**^** kg**^**−1**^**]**	r_sp_ = − 0.054 *p* = 0.587	r_sp_ =− 0.134 *p* = 0.179	r_sp_ =− 0.034 *p* = 0.735	r_sp_ =− 0.121 *p* = 0.222	r_sp_ =− 0.096 *p* = 0.337	r_sp_ =− 0.069 *p* = 0.492	r_sp_ =− 0.114 *p* = 0.251	r_sp_ =− 0.052 *p* = 0.600	r_sp_ =− 0.055 *p* = 0.584	r_sp_ =− 0.079 *p* = 0.429
	**V’E/V’CO**_**2**_**@VT**_**1**_ **[a.u]**	r_sp_ = 0.063 *p* = 0.530	r_sp_ = 0.112 *p* = 0.258	r_sp_ = 0.072 *p* = 0.468	r_sp_ = 0.129 *p* = 0.193	r_sp_ = 0.076 *p* = 0.448	r_sp_ = 0.073 *p* = 0.462	r_sp_ = 0.062 *p* = 0.532	r_sp_ =− 0.036 *p* = 0.715	r_sp_ = 0.153 *p* = 0.122	r_sp_ = 0.179 *p* = 0.071
	**CCF**_**max**_**(HR)** **[a.u]**	**r**_**sp**_** =− 0.223** *p* = **0.026**	**r**_**sp**_** =− 0.325** **p < 0.001**	**r**_**sp**_** =− 0.208** *p* = **0.038**	**r**_**sp**_** =− 0.296** *p* = **0.003**	**r**_**sp**_** =− 0.201** ***p* = 0.045**	**r**_**sp**_** =− 0.218** ***p* = 0.029**	r_sp_ =− 0.074 *p* = 0.466	r_sp_ =− 0.079 *p* = 0.437	**r**_**sp**_** =− 0.328** **p < 0.001**	**r**_**sp**_** =− 0.299** ***p* = 0.003**
	**CCF**_**max**_**(V’O**_**2pulm**_**)** **[a.u]**	r_sp_ = 0.000 *p* = 0.997	r_sp_ =− 0.079 *p* = 0.433	r_sp_ =− 0.025 *p* = 0.805	r_sp_ =− 0.003 *p* = 0.980	r_sp_ = 0.015 *p* = 0.879	r_sp_ = 0.039 *p* = 0.699	r_sp_ = 0.019 *p* = 0.849	r_sp_ = 0.022 *p* = 0.828	r_sp_ =− 0.220 *p* = 0.028	r_sp_ =− 0.129 *p* = 0.200
Exercise at the level of the VT_1_	**V’O**_**2**_**@VT**_**1**_ **[ml · min**^**−1**^** kg**^**−1**^**]**	r_sp_ =− 0.185 *p* = 0.061	r_sp_ =− 0.021 *p* = 0.833	r_sp_ =− 0.110 *p* = 0.268	r_sp_ = 0.016 *p* = 0.877	r_sp_ =− 0.136 *p* = 0.170	r_sp_ = 0.028 *p* = 0.778	r_sp_ =− 0.083 *p* = 0.406	r_sp_ =− 0.100 *p* = 0.315	r_sp_ =− 0.114 *p* = 0.253	r_sp_ = 0.067 *p* = 0.504
	**V’E/V’CO**_**2**_**@VT**_**1**_ **[a.u]**	**r**_**sp**_** = 0.243** ***p* = 0.013**	r_sp_ = 0.101 *p* = 0.311	r_sp_ = 0.163 *p* = 0.098	r_sp_ = 0.118 *p* = 0.236	**r**_**sp**_** = 0.217** ***p* = 0.028**	r_sp_ = 0.060 *p* = 0.551	r_sp_ = 0.121 *p* = 0.225	r_sp_ = 0.081 *p* = 0.416	**r**_**sp**_** = 0.216** ***p* = 0.029**	r_sp_ = 0.069 *p* = 0.489
	**CCF**_**max**_**(HR)** **[a.u]**	**r**_**sp**_** =− 0.298** ***p* = 0.003**	**r**_**sp**_** =− 0.243** ***p* = 0.015**	**r**_**sp**_** =− 0.230** ***p* = 0.021**	**r**_**sp**_** =− 0.199** ***p* = 0.049**	**r**_**sp**_** =− 0.241** ***p* = 0.016**	r_sp_ =− 0.175 *p* = 0.084	r_sp_ =− 0.104 *p* = 0.301	r_sp_ =− 0.086 *p* = 0.395	**r**_**sp**_** =− 0.344** **p < 0.001**	**r**_**sp**_** =− 0.210** ***p* = 0.037**
	**CCF**_**max**_**(V’O**_**2pulm**_**)** **[a.u]**	r_sp_ =− 0.168 p = 0.095	r_sp_ =− 0.062 p = 0.544	**r**_**sp**_** =− 0.231** **p = 0.021**	r_sp_ =− 0.097 p = 0.338	r_sp_ =− 0.171 p = 0.090	r_sp_ =− 0.049 p = 0.627	r_sp_ =− 0.041 p = 0.689	r_sp_ = 0.051 p = 0.613	**r**_**sp**_** =− 0.260** **p = 0.009**	r_sp_ =− 0.143 *p* = 0.159

Bold values indicate statistically significant correlations

*Abbreviations: a.u* arbitrary units, *V’O*_*2*_*@VT*_*1*_ Oxygen uptake at the first ventilatory threshold (103/105 valid measurements), *V’E/V’CO*_*2*_*@VT*_*1*_ ventilatory equivalent for CO_2_ at the first ventilatory threshold (103/105 valid measurements), *CCF*_*max*_*(HR)* maximum of the cross-correlation function between work rate and heart rate (99/105 valid measurements), *CCFmax(V’O2pulmu)​* Maximum of the cross-correlation function between work rate and oxygen uptake (99/105 valid measurements), *acc* acceleration

## Discussion

The primary aim of the present study was to assess the effects of acute bouts of exercise on static balance performance in older adults. Additionally, we investigated whether aerobic fitness was related to the magnitude of post-exercise balance impairments. We hypothesized that older adults with better aerobic fitness would experience less impairment in postural stability after exercise compared to those with lower fitness levels. Finally, we assessed whether postural responses and markers of aerobic fitness differed between older adults with and without a history of falls.

Regarding the first aim, our findings indicate that physical exertion performed at the level of or exceeding the VT_1_ was associated with transient impairments in several parameters of static postural control in older adults (Fig. 4). However, the walking-control appointment also showed significant changes in several sway parameters following the walking bouts with perturbations, indicating that perturbed walking itself can transiently influence subsequent static balance (Table 5). Accordingly, the later post-walking assessments during the VT_1_-exercise appointment (AW1_VT1_ and AW2_VT1_) should not be interpreted solely as recovery from the preceding VT_1_ exercise bout. The direct comparison with the corresponding post-walking measurements in the walking-control appointment (AW1_PW_ and AW2_PW_) showed no significant differences in post-walking changes, suggesting that the additional effect of VT_1_ exercise was not clearly distinguishable from the effect of the walking protocol at these later time points (Figure S2 and Table S3).

Regarding the second aim, aerobic fitness markers were not associated with the magnitude of the immediate exercise-induced change in postural sway. However, faster heart rate adaptability was associated with better overall balance control, particularly after exercise exceeding VT_1_. Thus, circulatory adaptability appeared to be related to overall postural control, but not to the magnitude of the acute exercise-induced deterioration in balance.

Regarding the third aim, no significant differences in aerobic fitness markers were observed between participants with and without a history of falling. In terms of postural control, no overall group differences in postural sway were found. However, the groups showed different response patterns across the resting and subsequent post-walking measurements. These group-dependent patterns emerged across both the walking-control (AW1_PW_, AW2_PW_) and VT_1_-exercise (AW1_VT1_, AW2_VT1_) appointments and were therefore not specific to the preceding VT_1_-exercise bout.

### Effect of an aerobic exercise bout on balance performance

Exercise at an intensity exceeding the VT_1_ during Appointment 1 resulted in a significant increase in postural sway parameters (acceleration range, RMS, mean velocity, and path length). As illustrated in Fig. 4 a-e, sway parameters consistently increased from baseline (R, BEE) to immediately after exercise (AsVT_1_) and decreased following a 5-min seated rest (A5M). Similar effects were detected when exercise was performed at the level of VT_1_, with significant increases in sway area, acceleration range, RMS, and path length as depicted in Fig. 4 f-j. However, the interpretation of the subsequent AW1_VT1_ and AW2_VT1_ measurements requires caution. These time points followed not only the VT_1_ exercise bout, but also the standardized perturbed walking protocol. The direct comparison with the walking-control appointment showed no significant Appointment x Condition interaction, indicating that post-walking changes in static balance did not differ significantly between the walking-control and VT_1_-exercise appointments. Although significant Condition x Group interactions were observed for several sway parameters, no Appointment x Condition x Group interaction was present (Supplementary Table S3). Thus, the group-dependent differences in postural response were not specific to the VT_1_-exercise appointment. The AVT_1_ sway measurement most directly reflects the immediate response to exercise at the level of VT_1_, whereas AW1_VT1_ and AW2_VT1_ likely reflect a combined effect of preceding exercise, perturbed walking, and short-term recovery. Interestingly, the walking-control appointment also showed condition effects in several sway parameters, suggesting that perturbed walking alone can transiently alter static balance in older adults. These effects appeared less pronounced than the immediate post-exercise response observed after the VT_1_ bout, but they underline that the walking with perturbations procedure itself seems to have an effect on sway parameters.

These observations are in line with earlier studies, demonstrating that acute aerobic activity transiently destabilizes balance performance in older adults. Across the literature, different exercise modalities have been employed to elicit an aerobic challenge and/or fatigue in older adults including a 6-min walk test [1], sit-to-stand until exhaustion [13], cycle ergometer [12, 35] and treadmill protocols with varying intensities and durations [7, 8, 26, 42]. Regardless of exercise modality, temporary impairments in postural control in older adults were reported. For example, Stemplewski et al. [35] observed significant increases in sway after 10 min of moderate cycling exercise that normalized after 15 min, while Donath et al. [7, 8] found that maximal endurance or high-intensity interval treadmill protocols impaired balance for 10 to 30 min. Even low-intensity walking challenges have shown destabilizing effects, with [42] reporting knee joint instability and interlimb asymmetry after 30–60 min of treadmill walking and Alias and Justine [1] showing reduced balance performance after a 6-min walk.

In summary, the aforementioned findings, together with the present study support the concept that acute physical exertion can transiently impair static balance performance in older adults. In the present study, the clearest exercise-specific effect was observed immediately after exercise exceeding VT_1_ and immediately after the individualized VT_1_ exercise bout. By contrast, post-walking assessments should be interpreted as reflecting the combined influence of prior exercise, perturbed walking, and recovery. The time course of recovery appears to vary by exercise modality, intensity, and study population, with our participants recovering faster than reported for cycling protocols, possibly reflecting differences in workload and the relatively high aerobic fitness of our study population.

### Relationship between aerobic fitness and postural sway

Another aim of this study was to determine whether aerobic fitness influences balance performance in older adults. The results indicate that the adaptability of the circulatory system, as reflected by CCF_max_(HR), was consistently associated with better overall postural control both before and after exercise. Significant inverse correlations between CCF_max_(HR) and sway parameters (sway area, sway range, RMS sway and path length) were observed at exercise intensities at and above the VT_1_, suggesting that faster circulatory adaptability is linked to better postural stability under both baseline and post-exertional conditions. These findings suggest that heart rate adaptability may be associated with maintaining postural stability when older adults are exposed to physical demands (Table 6). However, when specifically examining the magnitude of exercise-induced balance impairments (Δ_sway_), no significant associations were observed with aerobic fitness markers (V′O_2_/kg@VT_1_, V′E/V′CO_2_@VT_1_, CCF_max_(V’O_2pulm_) and CCF_max_(HR)—Table S2, Supplementary Material). Thus, while circulatory adaptability seems to be related to overall balance performance, it was not associated with the extent of balance deterioration following exercise.

Our results complement previous research, examining the relationship between aerobic fitness and balance in older adults. Stemplewski et al. [35], for instance, examined older men performing 10 min of moderate-intensity cycling at 60% of the predicted maximal heart rate and reported significant post-exercise increases in center of pressure velocity together with rises in blood lactate concentration. Crucially, they reported blood lactate levels to be positively correlated with changes in sway (r = 0.62–0.65, p < 0.01), suggesting that participants with greater lactate responses displayed larger postural impairments after moderate exercise. Since elevated lactate at moderate workloads, in the context of physical exertion, may reflect insufficient oxygen delivery and/or utilization by the exercising muscles, a stronger lactate response can be interpreted as a marker of lower aerobic capacity and of a higher reliance on anaerobic metabolism. As in our study, the balance between oxygen supply and demand during physical exertion is addressed. While Stemplewski et al. [35] captured this mismatch through lactate accumulation, in the present study submaximal CPET-derived markers of aerobic fitness, such as V’O_2_@VT_1_, V’E/V’CO_2_, and cardiorespiratory adaptability, were evaluated. The absence of correlations between aerobic fitness markers and Δ_sway_ in our study may be explained by the high aerobic fitness of our study population. The 17 participants enrolled in the study of Stemplewski et al. [35] were defined as community-dwelling older men aged 65–74 (mean age: 68.4 ± 2.9 years) in relatively good health, self-sufficient in daily living, and with no falls in the prior year. However, no CPET was performed i.e., no standardized aerobic fitness markers such as V’O_2max_ or V’O_2_@VT_1_ were reported. In the present study, aerobic fitness was objectively assessed via CPET in a larger sample. Given the lack of direct aerobic fitness data in the study by Stemplewski et al. [35], fitness comparability between the two studies was not possible.

The importance of aerobic fitness for balance has also been highlighted when assessing balance under resting conditions. Maciaszek [21] reported a significant association between higher aerobic capacity, as measured by the 6-min walk test, and less sway area on a posturographic platform. However, the included participants had low activity levels and had not engaged in structured exercise for at least five years, thereby representing a markedly different population from our study population. In addition, aerobic capacity was measured indirectly via a field test rather than by individualized CPET as in our study. Since walking test performance is strongly influenced by body weight and motivation, the actual physiological strain may have varied considerably between participants. Furthermore, while Maciaszek [21] demonstrated an association between aerobic fitness and postural control in the resting condition, our study focuses on postural sway after controlled exercise stress, thereby addressing the dynamic interplay between aerobic fitness and balance. Similar methodological distinctions apply to the study conducted by Zhao and Chung [43], who also reported a correlation between a higher aerobic capacity in older adults and a lower risk of falling. Notably, older adults with a history of falling were excluded from the study, again leading to a substantially different study population compared to the present study. Moreover, their outcome was fall risk, assessed with the Fall Risk Test on the Biodex Balance System, with their outcome focused on fall risk classification (via the Biodex stability index), rather than direct postural sway as in our study. Furthermore, aerobic capacity was measured using a 2-min step test within the Senior Fitness Test battery, which differs considerably from the CPET-based measures applied in the present study.

Building on this research focus, Silveira et al. [34] applied a treadmill-based CPET approach more closely aligned with our methodology, but with the crucial difference of determining V’O_2peak_, defined as the highest oxygen uptake achieved during the test when a true V’O_2max_ could not be confirmed, through a maximal graded test. Unlike our submaximal treadmill protocol, their approach captured the participants’ highest aerobic capacity, which may be more sensitive for identifying interindividual differences in cardiorespiratory performance. In the study by Silveira et al. [34], older adults with structural cardiovascular disease, classified in stages B or C according to American College of Cardiology/American Heart Association standards [16], were recruited from two cardiac rehabilitation centers and followed prospectively for 12 months. Participants who experienced falls during the observation period exhibited significantly lower V’O_2peak_ values than those who did not (V’O_2peak_: 14.6 vs 18.6 ml⋅min^−1^⋅kg^−1^), indicating that reduced aerobic capacity may help identify individuals at higher risk of falling in clinically heterogeneous populations. Furthermore, higher V’O_2peak_ values were significantly associated with better static balance performance and reduced center of pressure sway, even after adjusting for confounders such as age and BMI. However, their study population exhibited a wider range of fitness levels and included solely individuals with cardiovascular disease, potentially amplifying the relationship between aerobic fitness and balance.

In contrast, the participants in the present study were relatively homogeneous with high aerobic fitness overall, as evidenced by V’O_2_@VT_1_ values exceeding predicted norms and consistent with Weber’s functional Category A. This ceiling effect may have obscured more subtle relationships between aerobic capacity and postural control in our study population. Moreover, we assessed VT_1_ and circulatory adaptability as submaximal markers of aerobic fitness, reflecting the capacity to sustain everyday activities and the ability of the circulatory system to adapt to changing metabolic demands. These submaximal measures provide insight into how efficiently oxygen uptake, delivery, and utilization are matched during moderate exertion. These two methodological approaches emphasize different aspects of aerobic performance.

In summary, the available evidence indicates that post-exercise postural instability in older adults may be linked to a mismatch between oxygen supply and demand, whether it is demonstrated via lactate accumulation [35] or V’O_2peak_ [34]. However, in the present study, aerobic fitness markers were not associated with the magnitude of the immediate exercise-induced change in postural sway. While circulatory adaptability was associated with overall postural control, our findings do not support a direct association between aerobic fitness and the magnitude of acute exercise-induced balance deterioration.

### Comparison of aerobic fitness and balance performance according to fall history

We found no significant differences in the markers of aerobic fitness (V′O_2_/kg@VT_1_, V′_E_/V′CO_2_@VT_1_, CCF_max_(V’O_2pulm_) and CCF_max_(HR)) between the groups considering fall history (wFH vs. nFH, Table 2). Both groups consisted of fit and active older adults with high levels of V’O_2_@VT_1_. To contextualize this study population’s fitness level, we calculated reference values for V’O_2_@VT_1_ using the algorithm published within the Study of Health in Pomerania [10]. For women in our study population, the predicted V’O_2_@VT_1_ was calculated at 13.65 ml⋅min^−1^⋅kg^−1^ whereas the predicted V’O_2_@VT_1_ for men was calculated to be 14.55 ml⋅min^−1^⋅kg^−1^. The measured oxygen uptake values for our study population were notably higher than the predicted reference values. In women we measured a V’O_2_@VT_1_ of 20.78 ± 3.48 ml⋅min^−1^⋅kg^−1^ whereas in men we measured a V’O_2_@VT_1_ of 21.65 ± 3.88 ml⋅min^−1^⋅kg^−1^, which represents a study population with little to no impairment in aerobic capacity. This rather homogeneous and high fitness level may have limited the detection of group differences in our study. It remains an open question whether such differences would become more apparent in older adults with lower or more heterogeneous levels of aerobic fitness.

Regarding postural control, no significant overall main effect of Group was observed, indicating that participants wFH and nFH did not consistently differ in their overall level of postural sway across the examined conditions. However, the direct comparison of matched conditions across the walking-control and VT_1_-exercise appointments revealed significant Condition x Group interactions for acceleration range, RMS sway, mean velocity, and path length. These findings indicate that the pattern of change from rest through the first and second post-walking measurements differed between participants wFH and nFH despite the absence of an overall main effect of Group. The corresponding measurements were AW1_PW_ and AW2_PW_ in the walking-control appointment and AW1_VT1_ and AW2_VT1_ in the VT_1_-exercise appointment. Consistent with these interaction effects, inspection of the condition-specific values suggests differences in the temporal response between groups. Participants with a history of falling showed a more pronounced transient increase in several sway parameters, particularly following the first walking bout. Importantly, no significant Appointment x Condition x Group interaction was observed for any sway parameter. Therefore, these group-dependent response patterns were not specific to the preceding VT_1_-exercise bout and appear to reflect differences in the postural response to the subsequent walking and perturbation protocol more generally. These findings suggest that fall history may be associated more with differences in the postural response to physical challenge than with consistently greater postural sway. In addition to these statistically significant interaction effects, graphical trends suggested further differences in recovery dynamics (see Figure S1, Supplementary Material). The nFH group appeared to return toward baseline levels more rapidly across multiple balance parameters (sway area, acceleration range, RMS, mean velocity and path length), whereas participants with a history of falling generally displayed a slower or less complete recovery pattern. These apparent recovery differences should be interpreted descriptively, as the individual condition-specific group differences were not statistically significant. This pattern aligns with previous research suggesting that individuals with impaired postural control or fall history may exhibit diminished adaptability to physical exertion. These nuanced observations support the hypothesis that physiological differences in recovery dynamics may exist, although whether these differences translate into an increased risk of falling directly after moderate physical activity remains unclear.

## Strengths and limitations

One limitation of this study is that the study population consisted primarily of robust and physically fit older adults, which may limit the generalizability of the findings to more diverse or vulnerable populations. A further limitation is that the present analysis focused on static balance parameters, and it remains unclear how the observed post-exercise changes in postural sway translate to gait stability or actual fall occurrence in everyday life. In addition, although the walking-control appointment allowed a direct comparison with the VT_1_-exercise appointment, this comparison was limited to matched time points only. The immediate post-exercise measurement after exercise at the level of the first ventilatory threshold (AVT_1_) could not be included in this direct appointment comparison because no corresponding measurement existed in the walking-control appointment. Therefore, the direct comparison mainly informs post-walking balance behavior after the standardized walking and perturbation protocol, rather than the isolated immediate effect of VT_1_ exercise. Furthermore, the walking-control appointment was not a passive rest control condition, because participants also completed two perturbed walking bouts at individually preferred walking speed. Therefore, the observed post-walking sway changes may reflect the combined effects of walking, perturbation exposure and short-term recovery.

A major strength of our study lies in the use of an objective CPET. Even though V’O_2max_ was not obtained, parameters such as V’O_2_@VT_1_, V’E/V’CO_2_@VT_1,_ and CCF_max_(HR) present valuable markers of aerobic fitness. These markers are derived from standardized CPET procedures and can therefore be readily implemented in future studies using comparable protocols. Furthermore, the implementation of submaximal exercise at the level of VT_1_, reflecting real-life exertion levels, represents an additional strength of the study design. Additionally, during the VT_1_-exercise appointment, each participant completed an exercise bout tailored to their individual VT_1_, ensuring both physiological relevance and reproducibility for future studies using similar methods.

## Conclusions

Our findings show that physical exertion at the level of the VT_1_ may negatively impact postural stability in older adults, particularly immediately after the exercise bout. However, the direct comparison between the walking-control and VT_1_-exercise appointments showed no significant differences in post-walking balance changes between appointment conditions. Thus, the measurements following VT_1_ exercise (AW1_VT1_ and AW2_VT1_) likely reflect the combined influence of preceding exercise, perturbed walking, and short-term recovery. The observed inverse relationship between circulatory adaptability and postural control parameters before and after exercise highlights a potential physiological mechanism warranting further investigation. In our study population of older adults with high aerobic fitness, aerobic fitness markers were not associated with the magnitude of the immediate exercise-induced balance change. Participants with and without a history of falling did not differ in overall postural sway, but showed different response patterns across the post-walking measurements. These group-dependent patterns were observed across both appointments and therefore cannot be attributed specifically to preceding VT_1_-exercise.

It remains unclear how the observed impairments in static balance translate to dynamic balance control and fall risk during everyday movements or during sports. Future studies should examine more diverse study populations, including individuals with varying levels of aerobic fitness, to clarify how aerobic fitness and circulatory adaptability interact with balance control, and how these mechanisms relate to fall risk in everyday life.

## Supplementary Information

Supplementary Material 1.

Supplementary Material 2.

## Data Availability

The datasets generated and analyzed during the current study are not publicly available due to data protection and participat privacy considerations. Data are available from the corresponding author on reasonable request.

## Abbreviations

A5M: After 5 min of seated rest
ACF: Auto-correlation function
acc.: Acceleration
ANOVA: Analysis of variance
AsVT_1_: After exercise exceeding the first ventilatory threshold
a.u.: Arbitrary units
AVT_1_: After exercise at the level of the first ventilatory threshold
AW1_VT1_: Sway after the first session of perturbed walking following VT_1_ exercise
AW2_VT1_: Sway after the second session of perturbed walking following VT_1_ exercise
AW1_PW_: Sway after the first session of perturbed walking during the walking-control appointment
AW2_PW_: Sway after the second session of perturbed walking in the walking-control appointment
BEE: Before exercise while wearing CPET equipment / before exercise equipped
BMI: Body mass index
CCF_max_(HR): Maximum of the cross-correlation function between work rate and heart rate
CCF_max_(V’O_2pulm_): Maximum of the cross-correlation function between work rate and pulmonary oxygen uptake
COPD: Chronic obstructive pulmonary disease
CPET: Cardiopulmonary exercise testing
CPET-Eq: Cardiopulmonary exercise testing equipment
DRKS: German Clinical Trials Register
ECG: Electrocardiogram
eVT_1_: Exercise protocol at the VT_1_-exercise appointment
eT1: Exercise protocol at Appointment 1
HR: Heart rate
nFH: Participants without fall history
PRBS: Pseudo-random binary sequences
R: Resting condition
RMS: Root mean square
SD: Standard deviation
Short FES-I: Short Falls Efficacy Scale International
SPPB: Short Physical Performance Battery
sVT_1_: Exercise exceeding the first ventilatory threshold/supra VT_1_
TUG: Timed Up and Go
V’E/V’CO_2_@VT_1_: Ventilatory equivalent for carbon dioxide at the first ventilatory threshold
V’O_2_: Oxygen uptake
V’O_2_@VT_1_: Oxygen uptake at the first ventilatory threshold
V’O_2max_: Maximal oxygen uptake
V’O_2peak_: Peak oxygen uptake
VT_1_: First ventilatory threshold
VT_1_-exercise: Exercise at the level of the first ventilatory threshold
wFH: Participants with fall history
WR: Work rate
