# Supplementary material for: Effects of acute bouts of exercise and aerobic fitness on static balance in older adults
Source: BMC Sports Sci Med Rehabil. 2026 Nov 10;18:585. doi: 10.1186/s13102-026-02226-2 (PMC13756955; doi:10.1186/s13102-026-02226-2)
Supplement: Supplementary file 2 [file 13102_2026_2226_MOESM2_ESM.docx]

Postural Sway acceleration area [m2∙s-4]

|  | nFH | wFH |
| --- | --- | --- |
| BE | 0.078 | 0.07 |
| BEE | 0.159 | 0.093 |
| As  VT1 | 0.166 | 0.143 |
| A5M | 0.086 | 0.154 |

|  | nFH | wFH |
| --- | --- | --- |
| BE | 0.073 | 0.068 |
| AVT1 | 0.104 | 0.108 |
| AW1 | 0.085 | 0.097 |
| AW2 | 0.088 | 0.087 |

Postural sway acceleration range [m2·s-2]

|  | nFH | wFH |
| --- | --- | --- |
| BE | 0.508 | 0.485 |
| BEE | 0.631 | 0.526 |
| As  VT1 | 0.777 | 0.671 |
| A5M | 0.521 | 0.719 |

|  | nFH | wFH |
| --- | --- | --- |
| BE | 0.484 | 0.466 |
| AVT1 | 0.647 | 0.648 |
| AW1 | 0.523 | 0.564 |
| AW2 | 0.526 | 0.53 |

Postural sway acceleration RMS [m∙s-2]

|  | nFH | wFH |
| --- | --- | --- |
| BE | 0.094 | 0.089 |
| BEE | 0.113 | 0.102 |
| As  VT1 | 0.143 | 0.13 |
| A5M | 0.099 | 0.125 |

|  | nFH | wFH |
| --- | --- | --- |
| BE | 0.091 | 0.088 |
| AVT1 | 0.109 | 0.113 |
| AW1 | 0.095 | 0.108 |
| AW2 | 0.1 | 0.1 |

Postural sway acceleration mean velocity [m∙s-1]

|  | nFH | wFH |
| --- | --- | --- |
| BE | 0.184 | 0.173 |
| BEE | 0.234 | 0.191 |
| As  VT1 | 0.25 | 0.219 |
| A5M | 0.196 | 0.227 |

|  | nFH | wFH |
| --- | --- | --- |
| BE | 0.186 | 0.173 |
| AVT1 | 0.192 | 0.199 |
| AW1 | 0.181 | 0.218 |
| AW2 | 0.212 | 0.207 |

Postural sway acceleration path length [m∙s-2]

|  | nFH | wFH |
| --- | --- | --- |
| BE | 7.766 | 8.169 |
| BEE | 8.347 | 8.367 |
| As  VT1 | 12.492 | 12.704 |
| A5M | 8.68 | 10.377 |

|  | nFH | wFH |
| --- | --- | --- |
| BE | 8.031 | 8.298 |
| AVT1 | 11.089 | 11.885 |
| AW1 | 8.928 | 10.134 |
| AW2 | 8.708 | 9.507 |

*Figure S1: Group differences for postural sway parameters in different conditions in Appointment 1 (a, b, c, d, and e) and VT1-exercise appointment (f, g, h, i, and j). Abbreviations: VT_1_ – first ventilatory threshold, R – resting, BEE – before exercise equipped, AsVT_1_ – after exercise exceeding (supra – VT_1_) the first ventilatory threshold - equipped, A5M – after 5 minutes of rest – not equipped, AVT_1_ – after exercise at the level of the first ventilatory threshold, AW1_VT1_ – after first session of perturbed walking following exercise at the level of the first ventilatory threshold, AW2_VT1_ – after second session of perturbed walking following exercise at the level of the first ventilatory threshold, RMS: root mean square, nFH – participants without a history of falling, wFH – participants with a history of falling*

Postural Sway acceleration area [m2∙s-4]

|  | Gesamt |
| --- | --- |
| Postural.Sway.Acc.Sway.Area.m.2.s.4.GLO_T2_Sway_VP1 | 0.0682431 |
| Postural.Sway.Acc.Sway.Area.m.2.s.4.GLO_T2_Sway_NP1 | 0.0793765 |
| Postural.Sway.Acc.Sway.Area.m.2.s.4.GLO_T2_Sway_NP2 | 0.0879696 |

|  | ohne Sturzereignis | mit Sturzereignis |
| --- | --- | --- |
| Postural.Sway.Acc.Sway.Area.m.2.s.4.GLO_T2_Sway_VP1 | 0.0686104 | 0.0679167 |
| Postural.Sway.Acc.Sway.Area.m.2.s.4.GLO_T2_Sway_NP1 | 0.06835 | 0.0891778 |
| Postural.Sway.Acc.Sway.Area.m.2.s.4.GLO_T2_Sway_NP2 | 0.0793479 | 0.0956333 |

Postural sway acceleration range [m2·s-2]

|  | Gesamt |
| --- | --- |
| Postural.Sway.Acc.Range.m.s.2.GLO_T2_Sway_VP1 | 0.464735 |
| Postural.Sway.Acc.Range.m.s.2.GLO_T2_Sway_NP1 | 0.522167 |
| Postural.Sway.Acc.Range.m.s.2.GLO_T2_Sway_NP2 | 0.514098 |

|  | ohne Sturzereignis | mit Sturzereignis |
| --- | --- | --- |
| Postural.Sway.Acc.Range.m.s.2.GLO_T2_Sway_VP1 | 0.460875 | 0.468167 |
| Postural.Sway.Acc.Range.m.s.2.GLO_T2_Sway_NP1 | 0.479417 | 0.560167 |
| Postural.Sway.Acc.Range.m.s.2.GLO_T2_Sway_NP2 | 0.492333 | 0.533444 |

Postural sway acceleration RMS [m∙s-2]

|  | Gesamt |
| --- | --- |
| Postural.Sway.Acc.RMS.Sway.m.s.2.GLO_T2_Sway_VP1 | 0.0877255 |
| Postural.Sway.Acc.RMS.Sway.m.s.2.GLO_T2_Sway_NP1 | 0.0951686 |
| Postural.Sway.Acc.RMS.Sway.m.s.2.GLO_T2_Sway_NP2 | 0.0993118 |

|  | ohne Sturzereignis | mit Sturzereignis |
| --- | --- | --- |
| Postural.Sway.Acc.RMS.Sway.m.s.2.GLO_T2_Sway_VP1 | 0.0878521 | 0.087613 |
| Postural.Sway.Acc.RMS.Sway.m.s.2.GLO_T2_Sway_NP1 | 0.0887104 | 0.100909 |
| Postural.Sway.Acc.RMS.Sway.m.s.2.GLO_T2_Sway_NP2 | 0.0940875 | 0.103956 |

Postural sway acceleration mean velocity [m∙s-1]

|  | Gesamt |
| --- | --- |
| Postural.Sway.Acc.Mean.Velocity.m.s.GLO_T2_Sway_VP1 | 0.176082 |
| Postural.Sway.Acc.Mean.Velocity.m.s.GLO_T2_Sway_NP1 | 0.177954 |
| Postural.Sway.Acc.Mean.Velocity.m.s.GLO_T2_Sway_NP2 | 0.198311 |

|  | ohne Sturzereignis | mit Sturzereignis |
| --- | --- | --- |
| Postural.Sway.Acc.Mean.Velocity.m.s.GLO_T2_Sway_VP1 | 0.184067 | 0.168985 |
| Postural.Sway.Acc.Mean.Velocity.m.s.GLO_T2_Sway_NP1 | 0.166106 | 0.188485 |
| Postural.Sway.Acc.Mean.Velocity.m.s.GLO_T2_Sway_NP2 | 0.183979 | 0.21105 |

Postural sway acceleration path length [m∙s-2]

|  | Gesamt |
| --- | --- |
| Postural.Sway.Acc.Path.Length.m.s.2.GLO_T2_Sway_VP1 | 7.95029 |
| Postural.Sway.Acc.Path.Length.m.s.2.GLO_T2_Sway_NP1 | 8.85431 |
| Postural.Sway.Acc.Path.Length.m.s.2.GLO_T2_Sway_NP2 | 8.74235 |

|  | ohne Sturzereignis | mit Sturzereignis |
| --- | --- | --- |
| Postural.Sway.Acc.Path.Length.m.s.2.GLO_T2_Sway_VP1 | 7.60604 | 8.2563 |
| Postural.Sway.Acc.Path.Length.m.s.2.GLO_T2_Sway_NP1 | 8.00604 | 9.60833 |
| Postural.Sway.Acc.Path.Length.m.s.2.GLO_T2_Sway_NP2 | 8.27417 | 9.15852 |

*Figure S2: Graphic illustration of the effects of perturbed walking on static balance during the walking-control appointment for the whole study population a, b, c, d, and e. Group differences are shown in the graphs f, g, h, i, and j. Abbreviations: R – resting, AW1_PW_ – after first session of perturbed walking, AW2_PW_ – after second session of perturbed walking, RMS: root mean square, wFH – without fall history, nFH – participants without a history of falling, wFH – participants with a history of falling, * - p<0.05 and ** - p<0.001*

Table S1: Increase in postural sway for different variables expressed as Δ sway (postural sway after exercise minus postural sway before exercise).

|  | Δ acc. sway area  [m^2^∙s^-4^] | Δ acc. Range  [m^2^·s^-2^] | Δ acc. RMS  Sway  [m∙s^-2^] | Δ acc. mean velocity  [m∙s^-1^] | Δ acc. path length  [m∙s^-2^] |
| --- | --- | --- | --- | --- | --- |
| Appointment 1 (AsVT_1_) | 0.029 ± 0.367 | 0.144 ± 0.565 | 0.028 ± 0.076 | 0.022 ± 0.215 | 4.185 ± 4.678 |
| VT_1_-exercise appointment | 0.035 ± 0.067 | 0.172 ± 0.451 | 0.021 ± 0.037 | 0.016 ± 0.104 | 3.332 ± 3.535 |

*Abbreviations: Δ acc. sway – delta acceleration sway (Δ_acc.sway =_ sway_afterexercise_ – sway_beforeexercise_), AsVT_1_ – after exercise exceeding the VT_1_, AVT_1_ – after exercise at the level of VT_1_*

Table S2: Correlations between markers of aerobic fitness and balance changes (Δ_sway_) after exercise at the level of VT_1_ and after exercise exceeding the level of the VT_1_

|  | Δ acc. sway area  [m^2^∙s^-4^] | | Δ acc. sway range  [m^2^·s^-2^] | | Δ acc. sway RMS  [m∙s^-2^] | | Δ acc.  sway mean  velocity  [m∙s^-1^] | | Δ acc. sway path length  [m∙s^-2^] | |
| --- | --- | --- | --- | --- | --- | --- | --- | --- | --- | --- |
|  | **VT_1_** | **sVT_1_** | **VT_1_** | **sVT_1_** | **VT_1_** | **sVT_1_** | **VT_1_** | **sVT_1_** | **VT_1_** | **sVT_1_** |
| V’O_2_@VT_1_  [ml·min^-1^·kg^-1^] | r_sp_=0.082  p=0.414 | r_sp_=-0.090  p=0.368 | r_sp_=0.070  p=0.485 | r_sp_=-0.083  p=0.401 | r_sp_=0.098  p=0.325 | r_sp_=0.011  p=0.910 | r=0.001  p=0.991 | r_sp_=0.081  p=0.414 | r_sp_=0.119  p=0.232 | r_sp_=-0.048  p=0.630 |
| V’E/V’CO_2_@VT_1_  [a.u.] | r_sp_=0.018  p=0.861 | r_sp_=0.073  p=0.463 | r_sp_=0.035  p=0.728 | r_sp_=0.119  p=0.232 | r_sp_=-0.051  p=0.610 | r_sp_=0.026  p=0.794 | r=-0.016  p=0.870 | r_sp_=-0.078  p=0.436 | r_sp_=-0.053  p=0.594 | r_sp_=0.121  p=0.222 |
| CCF_max_(HR)  [a.u.] | r_sp_=-0.069  p=0.497 | r_sp_=-0.148  p=0.142 | r_sp_=-0.033  p=0.748 | r_sp_=0.162  p=0.106 | r_sp_=-0.007  p=0.942 | r_sp_=-0.078  p=0.441 | r=0.015  p=0.886 | r_sp_=0.095  p=0.322 | r_sp_=0.047  p=0.643 | r_sp_=-0.162  p=0.106 |
| CCF_max_(VO_2pulm_)  [a.u.] | r_sp_=0.077  p=0.449 | r_sp_=0.030  p=0.738 | r_sp_=0.072  p=0.477 | r_sp_=0.038  p=0.707 | r_sp_=0.079  p=0.434 | r_sp_=0.038  p=0.707 | r=0.003  p=0.715 | r_sp_=-0.009  p=0.928 | r_sp_=-0.001  p=0.996 | r_sp_=-0.031  p=0.758 |

*Abbreviations: a.u. – arbitrary units,* Δ_sway_ = value after exercise minus value before exercise, VT_1_ – exercise at the level of the first ventilatory threshold, sVT_1_ *– exercise exceeding the level of the first ventilatory threshold, V’O_2_@VT_1_ - Oxygen uptake at the first ventilatory threshold (103/105 valid measurements), V’E/V’CO_2_@VT_1_ - ventilatory equivalent for CO_2_ at the first ventilatory threshold (103/105 valid measurements), CCF_max_(HR) - maximum of the cross-correlation function between work rate and heart rate (99/105 valid measurements), CCF_max_(V’O_2pulm_) - maximum of the cross-correlation function between work rate and oxygen uptake (99/105 valid measurements), acc – acceleration, RMS: root mean square,*

Table S3: Direct comparison between the VT_1_-exercise and walking-control appointments across matched time points

|  | Group | Walking-control appointment | | | VT1-exercise appointment | | | Appointment | Condition | Group | Appointment x condition | Appointment x group | Condition x group | Appointment x condition x group |
| --- | --- | --- | --- | --- | --- | --- | --- | --- | --- | --- | --- | --- | --- | --- |
|  |  | **R** | **AW1_PW_** | **AW2_PW_** | **R** | **AW1_VT1_** | **AW2_VT1_** |  |  |  |  |  |  |  |
| Sway area  [m²·s^-4^] | Total | 0.068 ± 0.040 | 0.079  ±  0.055 | 0.087  ±  0.087 | 0.071 ± 0.050 | 0.092  ±  0.079 | 0.087  ±  0.065 | p=0.359 | **p<0.001** | p=0.320 | p=0.455 | p=0.516 | p=0.054 | p=0.714 |
|  | nFH | 0.069 ± 0.042 | 0.068  ±  0.044 | 0.079  ±  0.060 | 0.073 ± 0.052 | 0.078  ±  0.066 | 0.086  ±  0.074 |  |  |  |  |  |  |  |
|  | wFH | 0.068 ± 0.039 | 0.089  ±  0.062 | 0.096  ±  0.106 | 0.070 ± 0.050 | 0.099  ±  0.072 | 0.087  ±  0.053 |  |  |  |  |  |  |  |
| Acceleration range  [m^2^·s^-2^] | Total | 0.464 ± 0.133 | 0.522  ±  0.234 | 0.514  ±  0.211 | 0.475 ± 0.138 | 0.544  ±  0.221 | 0.528  ±  0.180 | p=0.367 | **p<0.001** | p=0.244 | p=0.969 | p=0.340 | **p=0.022** | p=0.942 |
|  | nFH | 0.461 ± 0.132 | 0.479  ±  0.156 | 0.492  ±  0.150 | 0.483 ± 0.147 | 0.501  ±  0.163 | 0.518  ±  0.184 |  |  |  |  |  |  |  |
|  | wFH | 0.478 ± 0.135 | 0.560  ±  0.283 | 0.533  ±  0.254 | 0.470 ± 0.133 | 0.565  ±  0.210 | 0.524  ±  0.153 |  |  |  |  |  |  |  |
| RMS sway  [m·s^-2^] | Total | 0.087 ± 0.025 | 0.095  ±  0.030 | 0.099  ±  0.040 | 0.089 ± 0.028 | 0.102  ±  0.041 | 0.100  ±  0.035 | p=0.156 | **p<0.001** | p=0.238 | p=0.435 | p=0.577 | **p=0.006** | p=0.413 |
|  | nFH | 0.088 ± 0.027 | 0.089  ±  0.028 | 0.094  ±  0.032 | 0.091 ± 0.030 | 0.093  ±  0.032 | 0.100  ±  0.040 |  |  |  |  |  |  |  |
|  | wFH | 0.088 ± 0.025 | 0.101  ±  0.031 | 0.104  ±  0.047 | 0.089 ± 0.027 | 0.109  ±  0.044 | 0.100  ±  0.030 |  |  |  |  |  |  |  |
| Mean velocity  [m·s^-1^] | Total | 0.176 ± 0.086 | 0.177  ±  0.082 | 0.198  ±  0.111 | 0.170 ± 0.092 | 0.200  ±  0.111 | 0.209  ±  0.128 | p=0.066 | **p=0.013** | p=0.477 | p=0.513 | p=0.877 | **p=0.026** | p=0.340 |
|  | nFH | 0.184 ± 0.088 | 0.166  ±  0.080 | 0.184  ±  0.092 | 0.187 ± 0.097 | 0.179  ±  0.090 | 0.211  ±  0.146 |  |  |  |  |  |  |  |
|  | wFH | 0.169 ± 0.086 | 0.188  ±  0.084 | 0.211  ±  0.125 | 0.174 ± 0.091 | 0.221  ±  0.123 | 0.210  ±  0.115 |  |  |  |  |  |  |  |
| Path length  [m·s^-2^] | Total | 7.950 ± 2.844 | 8.854  ±  3.778 | 8.742  ±  3.727 | 8.169 ± 3.220 | 9.554  ±  4.215 | 9.123  ±  3.630 | **p=0.041** | **p<0.001** | p=0.109 | p=0.262 | p=0.891 | **p=0.012** | p=0.981 |
|  | nFH | 7.606 ± 2.258 | 8.006  ±  2.792 | 8.274  ±  2.540 | 7.866 ± 2.569 | 8.638  ±  2.811 | 8.649  ±  2.938 |  |  |  |  |  |  |  |
|  | wFH | 8.256 ± 3.270 | 9.608  ±  4.366 | 9.159  ±  4.515 | 8.406 ± 3.663 | 10.216  ±  4.913 | 9.511  ±  4.181 |  |  |  |  |  |  |  |

*Abbreviations: R – resting, AW1_PW_ – after first session of perturbed walking, AW2_PW_ – after second session of perturbed walking, AW1_VT1_ – after the first session of perturbed walking following exercise at the first ventilatory threshold, AW2_VT1_ – after the second session of perturbed walking following exercise at the first ventilatory threshold*
